# Dietary Supplements and Oxidative Stress Management in Young Adults Following Intensive Exercise: A Systematic Review

**DOI:** 10.3390/sports14070285

**Published:** 2026-07-06

**Authors:** Vlassios Kakouris, Maria Piagkou, George Triantafyllou, Karolina Akinosoglou

**Affiliations:** 1 Department of Anatomy, School of Medicine, Faculty of Health Sciences, National and Kapodistrian University of Athens (UoA), 11527 Athens, Greece; vlassiosk@dent.uoa.gr (V.K.); mapian@med.uoa.gr (M.P.); georgerose406@gmail.com (G.T.); 2 School of Science and Technology, Hellenic Open University, 26335 Patras, Greece; 3 Department of Medicine, University of Patras, 26504 Rio, Greece; 4 Department of Internal Medicine and Infectious Diseases, University General Hospital of Patras, 26504 Rio, Greece

**Keywords:** oxidative stress, dietary supplements, intensive exercise, young adults, antioxidant supplementation, exercise physiology, athletes

## Abstract

Strenuous exercise is a well-established physiological stimulus that enhances muscular strength and hypertrophy but can also increase reactive oxygen species production, leading to oxidative stress (OS). Numerous studies have investigated whether dietary supplements can attenuate exercise-induced OS, yet findings remain inconsistent, and methodological quality varies. This systematic review aimed to synthesize current clinical evidence on dietary supplementation for OS management in young adults undergoing intensive exercise and to evaluate study methodology critically. The review was conducted in accordance with PRISMA 2021 guidelines and the Cochrane Risk of Bias (RoB 2.0) framework and was prospectively registered in PROSPERO. A comprehensive search of MEDLINE (PubMed), Scopus, Cochrane Central, ClinicalTrials.gov, OpenGrey, and ISRCTN identified interventional and observational human studies assessing supplementation and OS biomarkers. Forty-six studies met the inclusion criteria. The analysis revealed substantial heterogeneity in study design and reporting quality. Frequent methodological limitations included incomplete reporting of allocation concealment, participant and investigator blinding, examiner involvement, and deviations from intended interventions. Despite these limitations, several studies reported favorable effects of specific supplements on OS modulation and post-exercise recovery. Overall, the findings highlight widespread methodological shortcomings and emphasize the need for standardized trial designs, consistent biomarker selection, and transparent reporting. Well-designed, long-term randomized controlled trials are required to establish robust, evidence-based guidelines for dietary supplement use in managing exercise-induced OS in young adults.

## 1. Introduction

Oxidative stress (OS) is broadly defined as a state of imbalance between the production of reactive oxygen species (ROS) and the capacity of endogenous antioxidant systems to neutralize them, resulting in potential damage to lipids, proteins, and DNA when this balance is not restored. Regular physical exercise is widely recognized as a cornerstone of health, contributing to improvements in muscular strength, metabolic function, and cardiovascular fitness [1]. Resistance and endurance training, in particular, promote muscle hypertrophy and functional adaptation. However, the relationship between exercise and oxidative stress is intensity-dependent and modality-specific: while moderate-intensity exercise is generally associated with transient, adaptive increases in ROS that are well-managed by endogenous antioxidant systems, excessive intensity or volume—whether in aerobic endurance training or high-load resistance exercise—can significantly amplify ROS production and potentially overwhelm these defenses, leading to OS [2,3]. It is therefore important to recognize that not all exercise induces the same degree of oxidative challenge; real-world training programs typically employ a periodized mixture of intensities, and the OS burden experienced by any given athlete will reflect this variation rather than representing a uniformly maximal stimulus. This phenomenon reflects a physiological paradox: while moderate ROS production is essential for cellular signaling and exercise-induced adaptation, excessive accumulation can overwhelm antioxidant defenses and cause oxidative damage to lipids, proteins, and nucleic acids [4,5,6,7].

Exercise-induced OS has been associated with transient muscle damage, inflammation, altered redox signaling, and delayed recovery, especially following high-intensity or prolonged training sessions [8,9]. It is important to distinguish, however, between aerobic and resistance exercise modalities in this context: prolonged continuous aerobic exercise (typically exceeding 45–60 min at moderate-to-high intensity) tends to generate OS through mitochondrial and cardiovascular pathways, whereas high-load resistance training produces more localized, mechanically-mediated ROS elevation with distinct temporal patterns of oxidative marker elevation and recovery. These differences have direct implications for the interpretation of supplementation trials and must be considered when comparing findings across studies involving different exercise modalities. In response, the human body adapts by upregulating endogenous antioxidant systems, including superoxide dismutase, catalase, and glutathione-dependent enzymes [10]. Nevertheless, athletes and physically active individuals frequently seek additional protection through dietary supplements, aiming to enhance antioxidant capacity, accelerate recovery, and preserve performance [11].

Dietary supplements encompass a wide range of compounds, including vitamins, minerals (particularly selenium and zinc), fatty acids, amino acids, and plant-derived bioactive substances [12,13,14]. Selenium and zinc are essential trace elements involved in endogenous antioxidant defense, and disturbances in their homeostasis have been associated with increased oxidative stress and alterations in lipid peroxidation and glutathione metabolism [13,14]. Among these, polyphenols, omega-3 fatty acids, curcumin, quercetin, coenzyme Q10, and melatonin have been extensively studied for their potential to modulate exercise-induced OS [15,16,17,18,19,20,21]. However, findings across studies remain inconsistent. While some investigations report reduced oxidative biomarkers and improved recovery, others demonstrate minimal effects or even suggest that excessive antioxidant intake may blunt beneficial training adaptations by interfering with redox-sensitive signaling pathways [22,23,24]. These divergent hypotheses underscore an ongoing controversy regarding the efficacy, safety, and optimal use of antioxidant supplementation in physically active populations.

**Figure 1:**
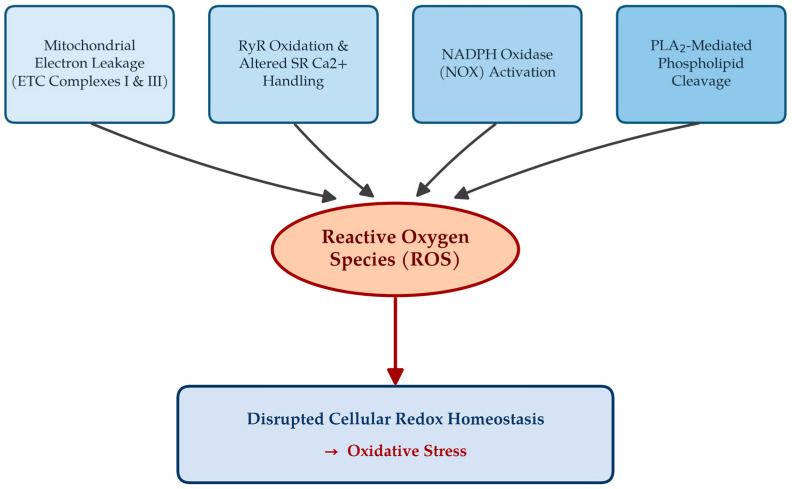
Revised schematic of proposed mechanisms of reactive oxygen species (ROS) production during intensive exercise in skeletal muscle, redrawn with enlarged labels for improved legibility. Four converging pathways are depicted: mitochondrial electron leakage at electron transport chain (ETC) complexes I and III; ryanodine receptor (RyR) oxidation with altered sarcoplasmic reticulum (SR) Ca^2+^ handling; NADPH oxidase (NOX) activation; and phospholipase A_2_ (PLA_2_)-mediated membrane phospholipid cleavage. Blue boxes represent the upstream ROS-generating mechanisms, the orange ellipse represents ROS, gray arrows indicate mechanistic convergence toward ROS production, and the red arrow denotes the downstream disruption of cellular redox homeostasis resulting in oxidative stress. These pathways collectively disrupt cellular redox homeostasis and promote oxidative stress.

At the cellular level, intensive exercise promotes ROS generation through several interconnected pathways, including mitochondrial electron leakage, ryanodine receptor oxidation with altered Ca^2+^ handling, NADPH oxidase activation, and phospholipase A_2_-mediated phospholipid cleavage (Figure 1) [5].

Despite a growing body of literature, variability in study design, supplement type and dosage, exercise protocols, and biomarker selection complicates interpretation and limits the development of evidence-based recommendations. Moreover, concerns have been raised regarding methodological quality, including incomplete reporting of randomization, blinding, and outcome assessment [25,26].

Therefore, the primary aim of this systematic review was to critically evaluate clinical studies examining dietary supplementation for the management of exercise-induced oxidative stress in young adults engaged in intensive exercise. In addition to synthesizing reported outcomes, this review assessed methodological quality and sources of bias using standardized frameworks. Beyond its academic contribution, this synthesis is intended to have direct practical relevance: clinicians, sports medicine practitioners, strength and conditioning coaches, and rehabilitation specialists are increasingly asked to advise physically active individuals on supplement use, and a critical appraisal of the existing evidence base can help inform more cautious, individualized recommendations in these settings [27]. The findings highlight both the potential of certain supplements to modulate oxidative stress and the substantial need for more rigorous, transparent, and standardized research to inform future clinical and athletic practice. Based on the available literature, we hypothesize that the evidence base will demonstrate heterogeneous effects of dietary supplementation on exercise-induced OS, with more consistent and robust findings emerging for polyphenol-rich and targeted ergogenic compounds than for broad-spectrum vitamin supplementation, and that methodological limitations—particularly inadequate blinding and inconsistent biomarker selection—will be a pervasive feature of the current evidence base, highlighting areas in need of improved research design.

## 2. Materials and Methods

### 2.1. Study Design and Registration

This systematic review was conducted in accordance with the Preferred Reporting Items for Systematic Reviews and Meta-Analyses (PRISMA) 2021 guidelines [28]. The review protocol was prospectively registered in the International Prospective Register of Systematic Reviews (PROSPERO; registration ID: 1014399), ensuring methodological transparency and adherence to predefined objectives and procedures. In the interest of full transparency as required by the PRISMA 2021 framework, we note the following items from the PRISMA 2021 checklist that were not applicable to this review or could not be fully implemented: (a) Item 15 (certainty assessment using GRADE) was applied at the supplement-category level rather than for individual outcomes, as the heterogeneity of biomarkers across studies precluded outcome-specific GRADE ratings; (b) Item 17 (effect estimates and confidence intervals) was not applicable, as the substantial heterogeneity precluded meta-analysis; (c) Item 22 (forest plots) was not produced for the same reason. All other applicable PRISMA 2021 items were addressed. The completed PRISMA 2021 checklist is available from the corresponding author upon request.

### 2.2. Eligibility Criteria

Studies were considered eligible if they met the following criteria: (i) involved human participants broadly described as young adults; no single internationally standardized age definition for this term exists in the sports and exercise science literature, so the applied age boundary of 17–45 years was derived empirically from the actual participant ages reported across the primary studies identified through our search, rather than imposed as an independent a priori external standard; (ii) included high-intensity aerobic and/or anaerobic exercise protocols; for aerobic exercise, this was operationally defined as sustained activity at ≥70% of maximal oxygen uptake (VO_2_max) for a minimum of 20 continuous minutes, consistent with established thresholds for exercise-induced oxidative stress; for resistance/anaerobic exercise, high intensity was defined as ≥80% of one-repetition maximum (1RM), or a Borg rating of perceived exertion (RPE) of ≥15; studies employing periodized or mixed-modality training were included where at least one component met these intensity thresholds, provided that the high-intensity component was the primary experimental stimulus; the exercise modality (aerobic, resistance, or mixed) and the session duration were extracted as study characteristics and are reported in Table 1; (iii) evaluated dietary supplement interventions intended to modulate oxidative stress; and (iv) reported biochemical or physiological oxidative stress outcomes, such as malondialdehyde (MDA), glutathione (GSH), catalase (CAT), or superoxide dismutase (SOD) activity. Randomized controlled trials, placebo-controlled trials, crossover studies, and observational studies were included. Exclusion criteria comprised animal studies, reviews, case reports, non-exercise-related investigations, and studies unavailable in full text or not published in English.

### 2.3. Information Sources and Search Strategy

A comprehensive literature search was conducted across MEDLINE (via PubMed), Scopus, Cochrane Central Register of Controlled Trials, ClinicalTrials.gov, OpenGrey, and ISRCTN. The search strategy combined controlled vocabulary and free-text terms for dietary supplementation, oxidative stress, young adults, and exercise, connected using Boolean operators. No restrictions were applied regarding publication year or study design. The final search was completed on 18 March 2025. Reference lists of included studies were also manually screened to identify additional eligible publications.

The full Boolean search equation applied to PubMed/MEDLINE was: (“dietary supplements”[MeSH] OR “dietary supplement”[tiab] OR “nutritional supplement*”[tiab] OR antioxidant*[tiab]) AND (“oxidative stress”[MeSH] OR “oxidative stress”[tiab] OR “reactive oxygen species”[tiab] OR ROS[tiab]) AND (“young adult”[MeSH] OR “young adults”[tiab] OR athletes[tiab]) AND (exercise[MeSH] OR exercise[tiab] OR training[tiab] OR “physical exertion”[tiab]). Equivalent search strings, adapted to the syntax of each database, are provided in the Supplementary Materials.

### 2.4. Study Selection

**Figure 2:**
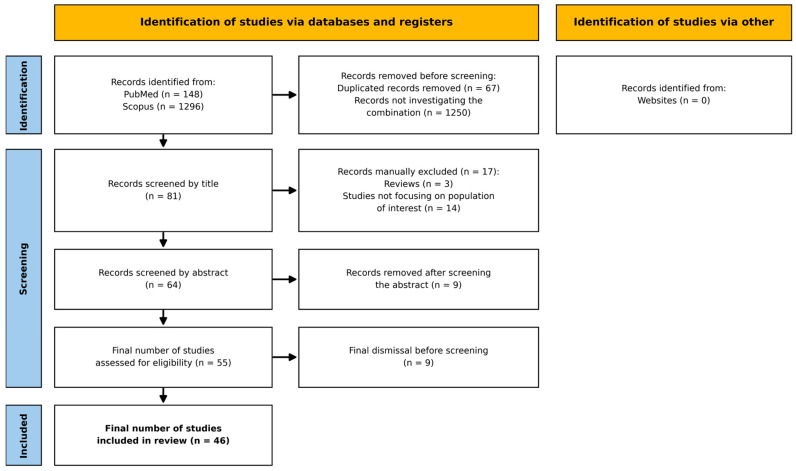
Flow diagram illustrating the process of study identification, screening, eligibility assessment, and inclusion, following PRISMA 2021 guidelines. A total of 1444 records were identified across electronic databases (PubMed: 148; Scopus: 1296). After removing duplicates (n = 67) and records unrelated to the inclusion criteria (n = 1250), 81 studies were screened by title, and 64 studies were screened by abstract. Following additional exclusions (n = 9) and final eligibility assessment (n = 55), a total of 46 studies met all inclusion criteria and were incorporated into the qualitative synthesis. No additional studies were retrieved from website sources or other registers.

All retrieved records were imported into Zotero (version 7.0.15), and duplicates were removed using both automated and manual procedures. Two reviewers independently screened titles and abstracts for eligibility. Full-text articles were subsequently assessed against the inclusion criteria. Disagreements were resolved through discussion and consensus. The study selection process followed PRISMA recommendations and is illustrated in the PRISMA flow diagram (Figure 2).

### 2.5. Data Extraction and Management

Data extraction was independently performed by two reviewers using a standardized data extraction form. Extracted variables included author information, publication year, study design, participant characteristics, exercise protocol, supplement type, dosage, and duration, outcome measures, and principal findings. Extracted data were cross-checked for accuracy, and discrepancies were resolved by mutual agreement. No automated data extraction tools were used.

### 2.6. Risk of Bias and Methodological Quality Assessment

Methodological quality was assessed using the Cochrane Risk of Bias (RoB 2.0) tool [29], evaluating five domains: randomization process, deviations from intended interventions, missing outcome data, outcome measurement, and selection of reported results. To enhance reporting clarity, outcomes were categorized into three tiers: specified, partially specified, and not specified. Assessments were conducted independently by both reviewers, followed by consensus discussions.

### 2.7. Certainty of Evidence

The Grading of Recommendations, Assessment, Development, and Evaluation (GRADE) approach [30] was applied to assess the overall certainty and strength of the evidence across studies, considering risk of bias, consistency, directness, and precision.

### 2.8. Ethical Considerations

Ethical approval was not required for this study, as it involved the analysis of previously published data. All included studies reported ethical approval and informed consent in accordance with their respective institutional requirements.

## 3. Results

### 3.1. Study Selection and Overview

#### 3.1.1. Identification and Inclusion of Studies

The study selection process is illustrated in the PRISMA flow diagram (Figure 2). A total of 1444 records were identified, including 148 records from electronic databases and 1296 records from manual searches and reference screening. After duplicate removal and eligibility screening, 46 studies met the inclusion criteria and were included in the qualitative synthesis.

#### 3.1.2. General Characteristics

The main characteristics of the included studies are summarized in Table 1. All studies examined dietary supplementation in young adults exposed to intensive aerobic, anaerobic, or mixed exercise protocols. Most studies used randomized, placebo-controlled, or crossover designs, although intervention duration, supplement type, and OS biomarkers varied considerably.

### 3.2. Effects of Dietary Supplements on Oxidative Stress

#### 3.2.1. Overall Findings

Across studies, dietary supplementation was frequently associated with modulation of exercise-induced OS, although results were heterogeneous.

Key reported effects included:

- Reductions in lipid peroxidation markers, particularly malondialdehyde (MDA);
- Increases in antioxidant enzyme activity, including superoxide dismutase (SOD), catalase (CAT), and glutathione-related enzymes;
- Attenuation of post-exercise inflammation and muscle damage markers in several trials.

#### 3.2.2. Divergent Outcomes

Contradictory findings were observed among studies investigating similar supplements. For example, omega-3 fatty acid (EPA/DHA) supplementation produced opposing outcomes across trials:

1. One study reported increased oxidative stress following supplementation.
2. Another reported reduction in oxidative and inflammatory biomarkers under comparable exercise conditions.

These differences reflect variability in study design, supplementation protocols, participant characteristics, and biomarker selection.

### 3.3. Methodological Quality and Risk of Bias

Section 3.3.1, Section 3.3.2, Section 3.3.3 and Section 3.3.4 below report the four formal Cochrane RoB 2.0 domains that constitute risk of bias in the strict methodological sense (i.e., features of study design and conduct that could bias the estimate of effect). Within the RoB 2.0 framework, a domain is rated as ‘some concerns’ or ‘high risk’ only when there is substantive reason—based on what was reported—to believe that the design or execution of the study could have introduced systematic error into the effect estimate. In this review, cases where a relevant procedural detail was simply absent from the report were coded as ‘not specified’ rather than as a confirmed methodological failure; such cases raise concerns about transparency but do not in themselves constitute evidence that a bias actually occurred. Section 3.4, by contrast, reports a supplementary appraisal of broader reporting completeness (e.g., equipment specification, examiner disclosure) that falls entirely outside the RoB 2.0 framework and is presented separately so as not to be conflated with formal risk of bias.

#### 3.3.1. Randomization and Allocation Concealment

Most studies (89.1%) reported randomization. However, the description of allocation concealment procedures was frequently absent or incomplete in the published reports: this represents a gap in reporting transparency, and while it prevents a confident assessment of this RoB 2.0 sub-domain, it does not in itself confirm that concealment was inadequate.

- Specified in 19.6% of studies;
- Partially specified in 30.4%;
- Not specified in 50.0%.

This pattern raises a concern about potential selection bias that cannot be ruled out; it does not, however, confirm that inadequate concealment occurred in studies where the procedure was simply undescribed.

#### 3.3.2. Deviations from Intended Interventions

Participant blinding was fully reported in 58.7% of studies and partially reported in 32.6%. Investigator blinding and documentation of deviations due to contextual factors were frequently absent from published reports (76.9% of studies). Under RoB 2.0, the absence of such descriptions means this domain could not be rated with confidence; it does not automatically imply that unblinding or protocol deviations occurred, only that the published report does not allow this to be verified.

#### 3.3.3. Outcome Measurement

This domain showed the highest consistency. Over 90% of studies clearly described validated measurement methods for OS biomarkers, with minimal differences between intervention and control groups.

#### 3.3.4. Selection of Reported Results

Only 41.3% of studies explicitly reported adherence to a prespecified statistical analysis plan. Where this was absent, the RoB 2.0 domain for selection of reported results could not be fully evaluated; this represents a transparency limitation and a source of uncertainty rather than confirmed selective reporting.

### 3.4. Performance and Reporting Bias

Reporting completeness varied across studies:

- Equipment specifications were provided in 95.7% of studies;
- The number of examiners was not reported in any study;
- Premises conditions were fully described in 30.4%;
- Ethical approval and informed consent were reported in more than 89% of studies.

### 3.5. Cumulative Risk of Bias Classification

**Figure 3:**
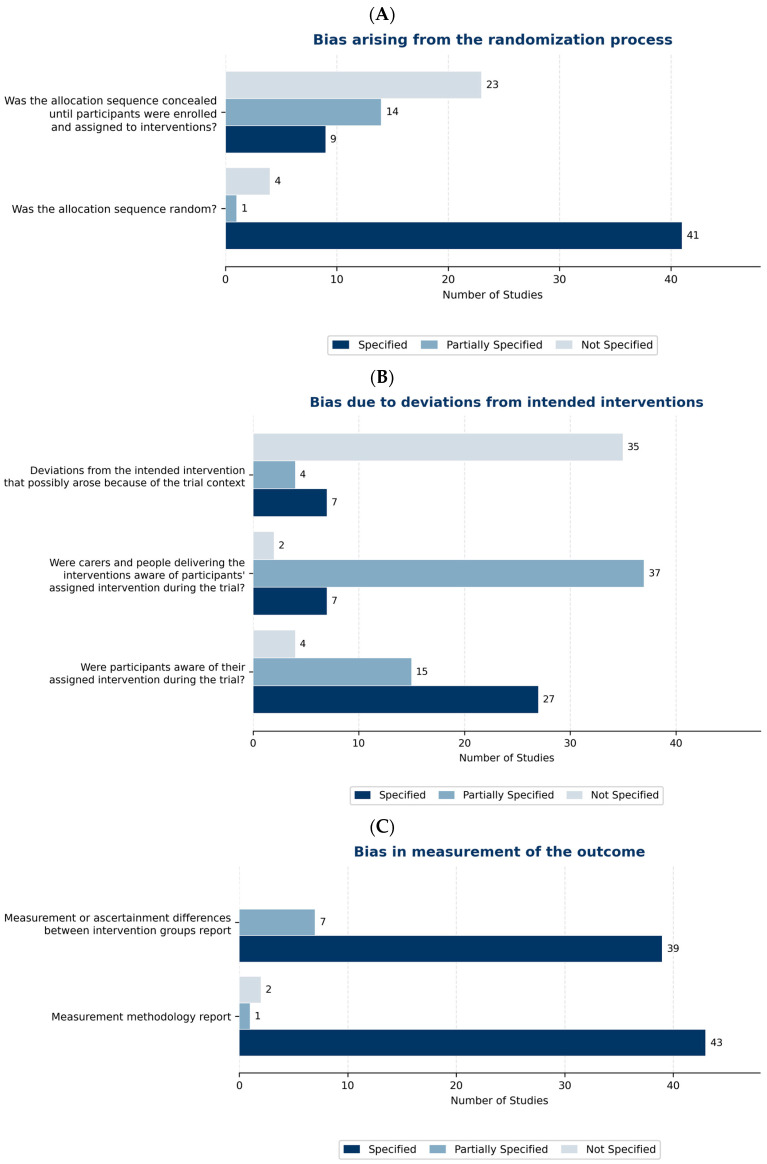

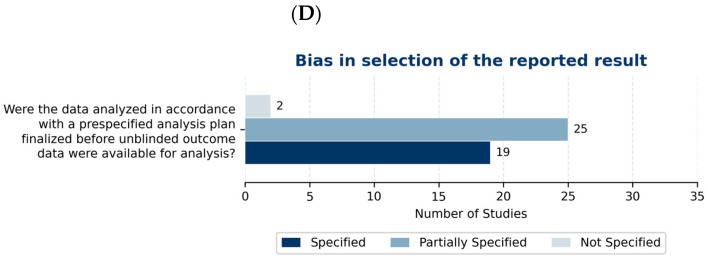
Risk-of-bias assessment of the included studies according to the Cochrane Risk of Bias 2.0 (RoB 2) tool [31,32,33,34,35,36,37,38,39,40,41,42,43,44,45,46,47,48,49,50,51,52,53,54,55,56,57,58,59,60,61,62,63,64,65,66,67,68,69,70,71,72,73,74,75,76]. (**A**) Bias arising from the randomization process, including sequence generation and allocation concealment. (**B**) Risk of bias due to deviations from intended interventions, including participant and investigator blinding and deviations from the intended intervention. (**C**) Risk of bias in the measurement of outcomes, including the validity and consistency of outcome assessment across intervention groups. (**D**) Risk of bias in the selection of the reported results, based on adherence to prespecified statistical analysis plans. Bars represent the number of studies in which each domain was classified as specified, partially specified, or not specified.

**Figure 4:**
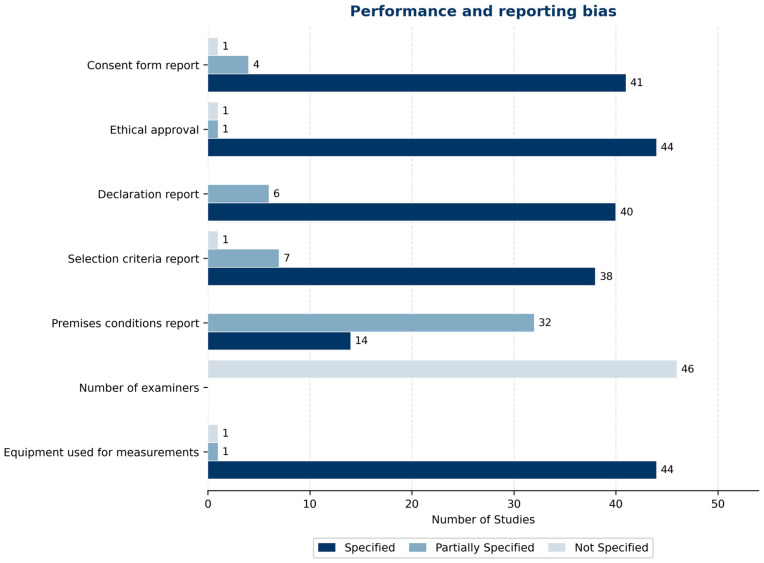
Performance and reporting bias across included studies, including equipment reporting, examiner disclosure, ethical approval, and informed consent. This figure addresses reporting completeness rather than formal RoB 2.0 risk of bias (see Section 3.3 introductory note).

**Figure 5:**
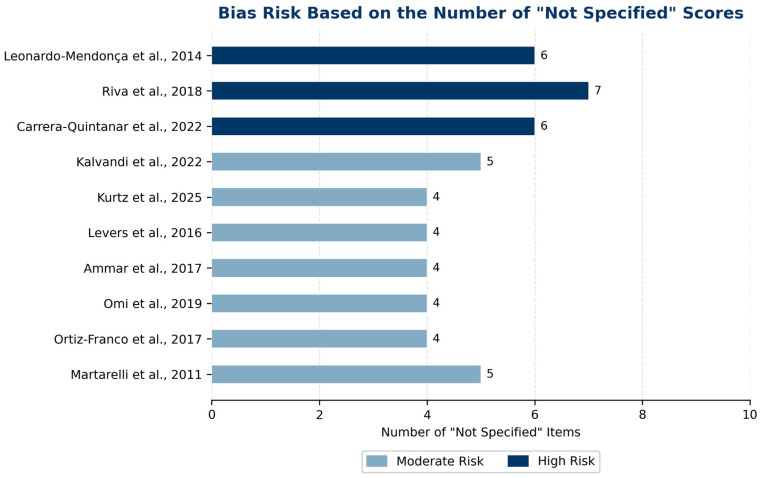
Distribution of studies according to the number of “Not Specified” methodological reporting items identified during the risk-of-bias assessment. Studies were categorized as moderate risk (light blue) or high risk (dark blue) based on the total number of reporting items classified as “Not Specified.” The figure includes the following studies: Martarelli et al. [41], Leonardo-Mendonça et al. [68], Levers et al. [55], Ammar et al. [54], Ortiz-Franco et al. [44], Riva et al. [67], Omi et al. [50], Carrera-Quintanar et al. [65], Kalvandi et al. [58], and Kurtz et al. [56]. The values shown at the end of each bar represent the total number of methodological reporting items classified as “Not Specified” for each study.

A cumulative tally of ‘not specified’ ratings—reflecting domains that could not be formally assessed due to absent reporting—was used as a secondary transparency indicator. On this basis, 10 studies were flagged as having moderate reporting incompleteness and three as having high reporting incompleteness, meaning that multiple RoB 2.0 domains could not be evaluated from the published record. The most frequently unassessable domains were allocation concealment, investigator blinding, and documentation of intervention deviations. It should be emphasized that this classification reflects the degree to which formal RoB 2.0 assessment was ‘blocked’ by absent reporting, not a conclusion that bias definitively occurred in these studies. Studies reporting few procedural details may have been rigorously conducted but inadequately reported; the classification therefore captures reporting quality, not confirmed methodological failure. For the formal RoB 2.0 domain-level results, see Figure 3A–D. Figure 4 and Figure 5 present the supplementary reporting-completeness and cumulative-transparency assessments respectively.

To summarize the interpretive principle applied throughout this section: within the Cochrane RoB 2.0 framework, a judgment of ‘some concerns’ or ‘high risk’ requires positive evidence that a design or conduct feature could have biased the result—not merely the absence of a description in the report. Where a relevant procedure was simply not described, this review assigned a ‘not specified’ code, which is recorded as a limitation of assessability rather than as a confirmed source of bias. Readers should therefore resist interpreting the ‘not specified’ tallies in this section as equivalent to a finding of methodological inadequacy. The formal RoB 2.0 domain judgments (Figure 3A–D), which were applied only where sufficient information existed to make a genuine assessment, should be regarded as the primary indicators of methodological risk. The reporting-completeness figures (Figure 4 and Figure 5) are supplementary transparency indicators and should be interpreted accordingly.

### 3.6. Summary of Experimental Conclusions

Overall, the included studies suggest that dietary supplementation may influence oxidative stress responses and recovery following intensive exercise in young adults. However, substantial methodological heterogeneity and reporting deficiencies limit the strength of experimental conclusions and underscore the need for standardized, rigorously designed trials.

## 4. Discussion

The primary objective of this systematic review was to synthesize current clinical evidence on the role of dietary supplements in managing exercise-induced oxidative stress (OS) in young adults and to evaluate the methodological quality of the available literature critically. The findings indicate that, although many studies report favorable effects of supplementation on oxidative stress biomarkers and recovery following intensive exercise, the evidence base is characterized by substantial heterogeneity and recurring methodological limitations.

### 4.1. Interpretation of Findings in the Context of Existing Literature

The present systematic review demonstrates that dietary supplementation may influence OS responses to intensive exercise in young adults [77]; however, the magnitude and consistency of these effects vary substantially across supplement categories. Overall, the findings support the working hypothesis that exogenous antioxidants can complement endogenous defense systems under conditions of elevated oxidative load, while also reinforcing emerging concerns about context-dependent, potentially maladaptive effects of supplementation.

Across the included studies, polyphenol-rich and plant-derived bioactive compounds—including tart cherry, blackcurrant, pomegranate, olive-derived phytocomplexes, and polyphenol-rich beverages—were associated with reductions in lipid peroxidation markers and improvements in post-exercise recovery in several studies; however, these findings should be interpreted cautiously given the generally small sample sizes and short intervention periods characterizing this body of evidence. These findings align with previous reports highlighting polyphenols’ capacity to modulate redox balance through both direct antioxidant activity and regulation of redox-sensitive signaling pathways. Nevertheless, isolated null findings within this category suggest that bioavailability, dosage, food matrix, and training status may critically influence outcomes. In contrast, omega-3 fatty acid supplementation (EPA/DHA) yielded contradictory results across studies, with both increases and decreases in oxidative stress reported. These opposing outcomes support the hypothesis that omega-3 effects may depend on baseline inflammatory status, exercise modality [78], and supplementation duration, underscoring the need for more standardized protocols.

Vitamin-based interventions, particularly vitamins C, D, and E, generally demonstrated limited or no efficacy in attenuating exercise-induced OS [79]. These findings are consistent with previous evidence suggesting that chronic high-dose vitamin supplementation may blunt training adaptations by disrupting redox-mediated signaling. Conversely, amino acid–related compounds (e.g., creatine, carnosine, anserine), probiotics, and selected ergogenic compounds (e.g., astaxanthin, coenzyme Q10, alpha-lipoic acid) showed generally favorable effects across the studies reviewed [80,81]. These findings should nonetheless be regarded as preliminary, as the supporting evidence remains constrained by small sample sizes, short intervention durations, and limited replication across independent research groups.

Beyond the broad categorization presented above, several methodological and physiological factors likely contribute to the divergent findings observed across the included studies, and a more explicit consideration of these moderators is warranted. Training status appears to be one such factor: habitually trained individuals typically exhibit upregulated endogenous antioxidant defenses relative to untrained or recreationally active participants, which may attenuate the apparent benefit of exogenous supplementation in already well-adapted athletes while producing more pronounced effects in less-trained populations (e.g., Prasertsri et al. [34], who directly compared trained and sedentary participants). Supplementation dosage and duration also varied considerably across studies, ranging from single acute pre-exercise doses to multi-week chronic protocols, and it is plausible that sub-threshold dosing or insufficiently long intervention periods account for several of the null findings reported (e.g., de Carvalho et al. [35]; Knab et al. [46]). Timing of supplementation relative to the exercise bout—whether administered before, during, or only after exercise—may further influence whether a given compound is bioavailable during the period of peak ROS production, although most included studies did not report this parameter in sufficient detail to allow systematic comparison. Finally, the heterogeneity in OS biomarkers selected across studies (e.g., MDA versus SOD versus total antioxidant capacity) means that apparently contradictory results may, in some cases, reflect genuinely different physiological endpoints rather than truly conflicting evidence regarding a single underlying construct [82]. Future primary research that systematically varies and reports these parameters would help disentangle their relative contribution to the heterogeneity observed in this field.

The potential for antioxidant supplementation to interfere with beneficial training adaptations, briefly introduced in Section 1, merits further consideration in light of the present findings. Moderate, exercise-induced ROS production is now recognized as a necessary signaling stimulus for several adaptive pathways, including activation of peroxisome proliferator-activated receptor gamma coactivator 1-alpha (PGC-1α)-mediated mitochondrial biogenesis and nuclear factor erythroid 2-related factor 2 (Nrf2)-dependent upregulation of endogenous antioxidant enzymes. High-dose or chronic antioxidant supplementation could, in principle, blunt these redox-sensitive signaling cascades, thereby attenuating the very training adaptations that intensive exercise is intended to produce. Although none of the included studies in the present review were specifically designed to test this hypothesis as a primary outcome, several findings are at least consistent with it: studies employing relatively high or prolonged antioxidant dosing protocols did not uniformly report superior outcomes compared with those using lower or more targeted doses, and in some cases (e.g., vitamin C and E co-supplementation [76]) reported no measurable benefit despite a plausible biochemical rationale. This—together with the broader literature on the antioxidant paradox—suggests that the relationship between supplementation and oxidative stress is unlikely to be linear, and that an indiscriminate “more is better” approach to antioxidant supplementation may not be appropriate for individuals seeking to preserve training-induced adaptations. We did not, however, perform a dose–response analysis within this review, and this remains an important avenue for future research rather than a conclusion that can be drawn directly from the included evidence.

A synthesis of supplement categories and overall evidence trends across included studies is provided in Table 2, which highlights areas of consensus, divergence, and uncertainty within the current literature. Collectively, these findings reinforce the concept that oxidative stress is not inherently detrimental but represents a tightly regulated physiological signal and that indiscriminate antioxidant supplementation may not uniformly benefit physically active populations.

### 4.2. Methodological Considerations and Sources of Bias

A key contribution of this review is the systematic evaluation of methodological quality using PRISMA 2021 and Cochrane RoB 2.0 frameworks. Two conceptually distinct types of limitation were identified across included studies and it is important not to conflate them. The first is genuine methodological concern: under the formal RoB 2.0 domains, several studies provided sufficient information to identify design or conduct features that could have introduced systematic error—most notably, unclear or absent allocation concealment in trials that described no concealment procedure whatsoever, and absence of any blinding where the design clearly precluded it. These constitute true sources of potential bias that should temper confidence in the reported effect estimates. The second is reporting incompleteness: the majority of the ‘not specified’ cases in this review reflect studies that did not describe certain procedural details in sufficient depth for the RoB 2.0 assessment to be completed, without there being positive evidence of a design flaw. These are properly interpreted as transparency limitations—they increase uncertainty but do not confirm bias. The two types of limitation are addressed separately in the Results (Section 3.3 and Section 3.4 respectively) and this distinction should be maintained when interpreting the Discussion.

Notably, outcome measurement showed the highest consistency, with most studies employing validated assays for OS biomarkers. In contrast, absent reporting of contextual factors—such as participant compliance, laboratory conditions, and examiner involvement—represents a gap in reporting transparency that increases uncertainty about internal and external validity, without in itself confirming that these factors were poorly controlled. These specific bias domains have direct implications for how the findings reported in Section 3.2 should be interpreted: studies with unclear allocation concealment or absent blinding are more susceptible to performance and detection bias, which may inflate apparently favorable supplement effects, whereas studies with incomplete reporting of deviations from intended interventions provide less certainty regarding whether the protocol as implemented matched the protocol as designed. Readers should therefore weigh the positive findings summarized in Table 2 in proportion to the RoB profile of the contributing studies (Section 3.3), rather than treating all reported effects as equally robust.

### 4.3. Broader Implications

From a broader perspective, these findings have implications beyond athletic performance, as OS is implicated in aging, inflammation, cardiovascular disease, and metabolic disorders [85]. Understanding how dietary supplements interact with exercise-induced redox responses may inform not only sports nutrition practices but also clinical strategies to manage oxidative stress in physically active and clinical populations. In practical terms, the heterogeneity and methodological limitations identified in this review suggest that clinicians, sports medicine practitioners, and coaches should avoid recommending high-dose or indiscriminate antioxidant supplementation as a routine adjunct to intensive training, particularly given the theoretical concern that such practices may blunt training adaptations (Section 4.1). Where supplementation is considered, the most consistent supporting evidence in this review relates to specific polyphenol-rich compounds and targeted ergogenic agents used at moderate doses over defined intervention periods, rather than broad-spectrum or high-dose antioxidant regimens. For rehabilitation settings specifically, where oxidative stress may already be elevated due to tissue injury or immobilization, an individualized approach that accounts for baseline antioxidant status, training history, and the specific clinical context is likely to be more appropriate than a uniform supplementation protocol. These practical considerations should, however, be regarded as provisional given the overall certainty of the evidence, and should be revisited as higher-quality trials become available. From a day-to-day coaching and sports medicine perspective, these findings suggest several actionable, if provisional, principles. First, regarding intensity modulation: given that the reviewed studies predominantly involved acute high-intensity bouts or sustained high-load protocols, practitioners should note that the oxidative stress burden—and therefore the theoretical rationale for supplementation—is likely most relevant during phases of high training load (e.g., pre-competition intensification blocks or high-volume endurance preparation), rather than during lower-intensity recovery or base-fitness phases where endogenous antioxidant systems are typically sufficient. Second, regarding the aerobic-to-resistance training ratio: the available evidence does not provide a basis for recommending a specific ratio of aerobic to resistance training to minimize OS, as the two modalities generate OS through different mechanisms and with different temporal profiles; however, combining both modalities within a periodized program—rather than relying on single-mode training—may help distribute the cumulative oxidative load and reduce the risk of exceeding antioxidant system capacity during any single session. Third, regarding dose-response: the reviewed literature does not consistently report dose-response data, but where it is available (e.g., curcumin [31,48], tart cherry [33,55], omega-3 [39,40,43]), the emerging pattern suggests that moderate doses within physiologically-relevant ranges tend to produce more reproducible effects than either very low or very high doses, and that chronic supplementation over several weeks generally produces more consistent OS attenuation than acute single-dose administration [76,86]. These practical observations remain preliminary and should be treated as directions for future investigation rather than established guidelines.

### 4.4. Future Research Directions

Future studies should prioritize rigorously designed, adequately powered, long-term randomized controlled trials with standardized exercise protocols and biomarker panels. Transparent reporting of randomization, blinding, examiner roles, and adherence to the intervention is essential. Additionally, research should explore dose–response relationships, timing of supplementation relative to exercise, and inter-individual factors such as sex, training status, and baseline antioxidant capacity.

### 4.5. Summary

In summary, while dietary supplementation shows potential for modulating exercise-induced oxidative stress in young adults, current evidence remains constrained by methodological heterogeneity and reporting deficiencies. Addressing these limitations is critical for establishing evidence-based guidelines and for clarifying when, how, and for whom antioxidant supplementation may be beneficial.

#### Strengths and Limitations

This systematic review has several notable strengths. First, it adhered strictly to the PRISMA 2021 guidelines and was prospectively registered in PROSPERO, enhancing methodological transparency and reducing the risk of selective reporting. Second, a comprehensive and sensitive search strategy was applied across multiple bibliographic and grey-literature databases, minimizing publication bias and increasing the likelihood of capturing all relevant evidence. Third, the review uniquely focused on young adults engaged in intensive exercise, a population frequently underrepresented in clinical reviews despite high supplement use. Fourth, methodological quality was evaluated using the Cochrane RoB 2.0 framework, supplemented by a structured three-tier reporting classification, allowing for a nuanced appraisal of reporting transparency and reproducibility. Finally, the inclusion of a wide range of supplement categories enabled a broad comparative perspective on antioxidant strategies.

Despite these strengths, several limitations must be acknowledged. Considerable heterogeneity in study design, supplement type, dosage, intervention duration, exercise protocols, and oxidative stress biomarkers precluded quantitative meta-analysis and necessitated a qualitative synthesis. Notably, the inclusion of both randomized controlled trials and non-randomized or observational studies within the same synthesis further increased this heterogeneity, as these designs differ substantially in their susceptibility to confounding, selection bias, and effect estimation; this should be borne in mind when interpreting the overall directionality of findings across the included literature. Many studies were characterized by small sample sizes and short intervention periods, limiting statistical power and the ability to infer long-term effects. Methodological weaknesses, remarkably incomplete reporting of allocation concealment, examiner involvement, and deviations from intended interventions, were recurrent and may have introduced bias. Additionally, the restriction to English-language publications may have resulted in language bias. Finally, variability in biomarker limitations and analytical methods limits cross-study comparability and hinders the establishment of standardized evidence-based recommendations.

Overall, while this review provides a robust synthesis of current evidence, these limitations highlight the need for larger, longer, and methodologically rigorous randomized controlled trials employing standardized oxidative stress outcomes and transparent reporting practices.

## 5. Conclusions

This systematic review provides a comprehensive synthesis of clinical evidence on the role of dietary supplements in managing exercise-induced OS among young adults engaged in intensive training. The findings suggest that certain supplement categories—including polyphenols, omega-3 fatty acids, curcumin, quercetin, and coenzyme Q10—may, under some conditions and in some populations, be associated with attenuation of exercise-induced oxidative stress markers; however, these signals are inconsistent across studies, derived largely from small and heterogeneous trials, and cannot at this stage be considered sufficient to form the basis of generalizable recommendations. However, the overall evidence remains heterogeneous and frequently limited by methodological shortcomings.

Critical weaknesses were identified across studies, particularly in the reporting of allocation concealment, blinding procedures, examiner involvement, and deviations from intended interventions. These limitations substantially reduce confidence in the reproducibility and generalizability of reported outcomes, and in combination with the extreme heterogeneity of supplements, doses, exercise modalities, participant populations, and oxidative stress biomarkers across the included literature, they preclude any strong or uniform conclusions about the efficacy of dietary supplementation for OS management following intensive exercise.

Taken together, the results suggest that dietary supplementation may hold some promise as a strategy for managing oxidative stress in physically active young adults; however, given the heterogeneity, modest sample sizes, and methodological limitations identified, current evidence is insufficient to support specific dosing recommendations or uniform clinical or athletic practice guidance at this time. Future research should prioritize well-designed, long-term randomized controlled trials with standardized exercise protocols—clearly specifying both modality and intensity—consistent oxidative stress biomarkers, explicit dose-response characterization, and transparent methodological reporting. Only through such research can robust, evidence-based guidelines for the safe and considered use of dietary supplements be developed for both athletic and clinical settings.

## Figures and Tables

**Table 1:** Characteristics of Included Studies Investigating the Effects of Dietary Supplements on Exercise-Induced Oxidative Stress in Young Adults, M-males, F-females.

No.	Study	Supplement(s) Examined	Study Design	Population Size	Overall Outcome *
1	Bai et al., 2023 [31]	Curcumin	Non-randomized prospective cohort	21 M, 7 F	+
2	Alkhatib et al., 2020 [32]	Anserine	Randomized, crossover, placebo-controlled trial	10 M	+
3	Hooper et al., 2021 [33]	Tart cherry extract	Randomized, crossover, placebo-controlled study	13 M	+
4	Prasertsri et al., 2019 [34]	Cashew apple juice	Randomized, double-masked, crossover study	10 trained M, 10 sedentary M	+
5	de Carvalho et al., 2019 [35]	Cocoa flavanols	Randomized, double-blind study	13 M	No effect
6	Hunt et al., 2021 [36]	Blackcurrant extract	Double-masked randomized trial	27 mixed	+
7	Rahimi, 2011 [37]	Creatine	Randomized, double-blind, placebo-controlled study	27 M	+
8	Martorell et al., 2015 [38]	DHA	Double-masked randomized study	15 M	+
9	Filaire et al., 2010 [39]	EPA/DHA	Randomized, double-blind study	20 M	No effect
10	Buonocore et al., 2020 [40]	n-3 fatty acids	Pilot study	21 trained, 18 sedentary	+
11	Martarelli et al., 2011 [41]	*L. rhamnosus* & *L. paracasei*	Single-blind study	24 M	+
12	Baralic et al., 2015 [42]	Astaxanthin	Randomized, double-masked study	40 M	+
13	Bloomer et al., 2009 [43]	EPA/DHA	Randomized, placebo-controlled crossover study	14 M	+
14	Ortiz-Franco et al., 2017 [44]	Melatonin	Randomized, double-blind, placebo-controlled trial	14 M	+
15	Kritikos et al., 2021 [45]	Whey vs. soy protein	Randomized controlled trial	10 M	+
16	Knab et al., 2013 [46]	Flavonoid-rich juice	Randomized crossover study	9 M	No effect
17	Stankiewicz et al., 2021 [47]	Chokeberry juice	Double-masked randomized trial	20 M	No effect
18	Takahashi et al., 2014 [48]	Curcumin	Double-blind, placebo-controlled crossover study	10 M	+
19	Braakhuis et al., 2014 [49]	Vitamin C/anthocyanins	Randomized three-treatment crossover	23 F	BC +
20	Omi et al., 2019 [50]	EMIQ-enriched protein	Randomized, double-blind trial	40 M	+
21	Cieślicka et al., 2022 [51]	Bovine colostrum	Placebo-controlled clinical trial	20 F	+
22	Shirai et al., 2023 [52]	Maslinic acid	Randomized, double-masked crossover trial	12 M	+
23	Withee et al., 2017 [53]	MSM	Randomized, double-masked trial	17 F, 5 M	No effect
24	Ammar et al., 2017 [54]	Pomegranate juice	Before-and-after study	9 M	+
25	Levers et al., 2016 [55]	Tart cherry powder	Randomized, double-masked, placebo-controlled study	18 M, 9 F	+
26	Kurtz et al., 2025 [56]	Quercetin + citrulline	Randomized, double-masked, placebo-controlled study	42 M, 8 F	No effect
27	Sadowska-Krępa et al., 2017 [57]	Ginkgo biloba	Randomized, double-masked trial	18 M	+
28	Kalvandi et al., 2022 [58]	Vitamin D_3_	Randomized, double-masked, placebo-controlled study	40 M	No effect
29	Williamson et al., 2018 [59]	Barley–wheat grass juice	Randomized crossover study	10 M	No effect
30	Tsao et al., 2023 [60]	Garlic	Single-masked crossover study	11 M	+
31	Lee et al., 2019 [61]	Octacosanol	Double-masked parallel study	26 M	+
32	Słowińska-Lisowska et al., 2014 [62]	L-carnosine	Randomized crossover study	14 M	+
33	Popovic et al., 2015 [63]	Vitamin C	Non-randomized two-group study	30 sedentary M, 30 athlete M	Partial +
34	Chaouachi et al., 2022 [64]	Spirulina	Randomized, double-masked trial	17 adults	+
35	Carrera-Quintanar et al., 2022 [65]	Polyphenol beverages	Three-arm study	30 M	+
36	Arent et al., 2010 [66]	Nutritional supplementation	Masked placebo-controlled study	24 M	+
37	Riva et al., 2018 [67]	Quercetin phytosome^®^	Self-selection experimental study	23	+
38	Leonardo-Mendonça et al., 2014 [68]	Dietary scheme only	Multi-phase study	10 M	No supplement effect
39	Ovchinnikov et al., 2022 [69]	Royal jelly + CoQ10	Randomized, double-masked trial	20 M	+
40	Huang et al., 2019 [70]	L. plantarum PS128	Double-masked experimental study	34 total	+
41	Salicio et al., 2016 [71]	Caffeine	Randomized, double-masked crossover study	24	+
42	Isenmann et al., 2020 [72]	Alpha-lipoic acid	Randomized crossover trial	17 M	+
43	Roberts et al., 2022 [73]	Olive-derived phytocomplex	Randomized crossover study	15	+
44	Gholami et al., 2021 [74]	Tomato powder	Randomized crossover study	11 M	+
45	Cases et al., 2017 [75]	PerfLoad^®^ polyphenols	Randomized crossover trial	15 M	+
46	Morrison et al., 2015 [76]	Vitamin C + E	Randomized, double-masked trial	11 M	No effect

* The Overall Outcome column denotes the directionality of change in the principal oxidative stress biomarker(s) reported by each study (“+” = statistically significant favorable change in at least one biomarker; “No effect” = no statistically significant change detected). This binary classification necessarily simplifies findings from studies that measured multiple biomarkers with divergent or partial responses (e.g., a reduction in lipid peroxidation without a corresponding change in antioxidant enzyme activity); such nuances are addressed narratively in Section 3.2 and Section 4.1, and biomarker-specific detail is not intended to be fully captured by this summary column.

**Table 2:** Summary of Dietary Supplement Categories and Their Effects on Exercise-Induced Oxidative Stress in Young Adults.

Supplement Category	Specific Supplements Examined	Number of Studies	Overall Evidence Trend	Key Observations
**Polyphenols & Plant Bioactives**	Tart cherry, blackcurrant, chokeberry, pomegranate, cocoa flavanols, olive-derived phytocomplex, tomato powder, PerfLoad^®^, polyphenol beverages	12	Mostly positive	Consistent reductions in oxidative biomarkers and improved recovery; some null effects (e.g., cocoa, chokeberry) suggest dose- and matrix-dependence
**Omega-3 Fatty Acids**	EPA, DHA, n-3 fatty acids	4	Mixed	Contradictory findings were reported, with both increased and reduced OS observed across studies.
**Curcumin & Derivatives**	Curcumin, maslinic acid	3	Positive	Repeated evidence of reduced OS markers and improved recovery [83] following intensive exercise
**Vitamins (C, D, E)**	Vitamins C, D_3_, C + E	5	Mostly no effect	Isolated or combined vitamin supplementation generally failed to attenuate OS; partial effects were observed in specific populations.
**Amino Acids & Related Compounds**	Creatine, L-carnosine, anserine	3	Positive	Improvements in antioxidant status and reduced exercise-induced oxidative damage.
**Protein-Based Supplements**	Whey protein, soy protein, EMIQ-enriched protein	3	Positive	Enhanced antioxidant capacity when combined with bioactive compounds
**Probiotics & Fermented Products**	Lactobacillus spp., L. plantarum PS128, bovine colostrum	4	Positive	Improved redox balance and inflammatory modulation; evidence limited by small samples
**Ergogenic & Miscellaneous Compounds**	Astaxanthin, CoQ10, royal jelly, caffeine, garlic, alpha-lipoic acid, octacosanol	7	Mostly positive	Beneficial effects reported, but heterogeneity in protocols limits generalizability [80,81,84].
**Dietary Patterns/No Supplement**	Specific dietary scheme only	1	No supplement effect	Adequate diet alone is sufficient to maintain redox balance in trained individuals.

## Data Availability

No new data were created or analyzed in this study. All data supporting the findings of this systematic review are contained within the published articles included in the review and their Supplementary Materials. Additional extracted data are available from the corresponding author upon reasonable request.

## Supplementary Materials

The following supporting information can be downloaded at: https://www.mdpi.com/article/10.3390/sports14070285/s1.

## Abbreviations

The following abbreviations are used in this manuscript:

**Abbreviation**	**Meaning**
ATP	adenosine triphosphate
CAT	catalase
CK-MB	creatine kinase myocardial band
CoQ10	coenzyme Q10
DSHEA	Dietary Supplement Health and Education Act
DHA	docosahexaenoic acid
DNA	deoxyribonucleic acid
EPA	eicosapentaenoic acid
ETC	electron transport chain
FADH_2_	flavin adenine dinucleotide (reduced form)
GOT	glutamic-oxaloacetic transaminase
GPT	glutamate-pyruvate transaminase
Gpx	glutathione peroxidase
GSH	glutathione
GRADE	Grading of Recommendations, Assessment, Development, and Evaluation
MDA	malondialdehyde
MSM	methylsulfonylmethane
NADH	nicotinamide adenine dinucleotide (reduced form)
NOX	NADPH oxidase
OS	oxidative stress
PLA_2_	phospholipase A_2_
PRISMA	Preferred Reporting Items for Systematic Reviews and Meta-Analyses
PROSPERO	International Prospective Register of Systematic Reviews
RCT	randomized controlled trial
RBC	red blood cell
RoB	risk of bias
ROS	reactive oxygen species
RyR	ryanodine receptor
SOD	superoxide dismutase
SR	sarcoplasmic reticulum
TAC	total antioxidant capacity
TT	transverse tubules
WBC	white blood cell
**BC**	blackcurrant
**EMIQ**	enzymatically modified isoquercitrin
