# Supplementary material for: Dietary Supplements and Oxidative Stress Management in Young Adults Following Intensive Exercise: A Systematic Review
Source: Sports (Basel). 2026 Jul 6;14(7):285. doi: 10.3390/sports14070285 (PMC13419206; doi:10.3390/sports14070285)
Supplement: Supplementary file 1 [file sports-14-00285-s001.zip › sports-4370491-supplementary.pdf]

PRISMA 2021 CHECKLIST

**Manuscript Title:**	Dietary Supplements and Oxidative Stress Management in Young Adults Following Intensive Exercise: A Systematic Review
**Journal:**	Sports (MDPI)
**Review Type:**	Systematic Review (Qualitative Synthesis)
**PROSPERO Registration:**	CRD42024014399

Note on non-applicable items: This review is a qualitative systematic synthesis; no meta-analysis was conducted. Items requiring effect estimates, forest plots, or pooled confidence intervals (Items 12, 13d, 13e, 13f, 20b, 20c, 20d) are therefore not applicable (N/A). Item 15 (certainty assessment) was applied at the supplement-category level rather than for individual outcomes due to heterogeneity of biomarkers across studies. Items 10b and 16b were addressed where relevant. N/A items are highlighted in the table below.

Section	Item No.	PRISMA 2021 Checklist Item	Location in Manuscript	Notes / Comments
TITLE	1	Identify the report as a systematic review.	Title; Article type field (p. 1)	Manuscript title explicitly contains "Systematic Review"; article type field states "Review".
ABSTRACT	2	See the PRISMA 2020 for Abstracts checklist.	Abstract (p. 1)	Abstract reports: background, methods (search strategy, eligibility, RoB framework, PROSPERO registration), results (n=46 studies, key findings), and conclusions. PRISMA for Abstracts items addressed.
INTRODUCTION	3	Describe the rationale for the review in the context of existing knowledge.	Section 1. Introduction	Sections 1 (¶1–5) describe the physiological rationale (exercise-induced OS, ROS mechanisms), the clinical and sports-nutrition context, and gaps in the existing literature.
	4	Provide an explicit statement of the objective(s) or question(s) the review addresses.	Section 1. Introduction (final ¶)	Explicit aim statement: "the primary aim of this systematic review was to critically evaluate clinical studies examining dietary supplementation for the management of exercise-induced oxidative stress in young adults engaged

				in intensive exercise." Research hypothesis also stated.
**METHODS**	**5**	Specify the inclusion and exclusion criteria for the review and how studies were grouped for the syntheses.	Section 2.2. Eligibility Criteria	Four inclusion criteria defined (population: young adults 17–45 y; intervention: dietary supplements; exercise: high-intensity aerobic ≥70% VO_2max/≥20 min or resistance ≥80% 1RM; outcomes: biochemical OS markers). Exclusion criteria listed. Studies grouped by supplement category in Table 2.
	**6**	Specify all databases, registers, websites, organisations, reference lists and other sources searched or consulted. Specify the date when each source was last searched.	Section 2.3. Information Sources and Search Strategy	Six sources searched: MEDLINE (PubMed), Scopus, Cochrane Central, ClinicalTrials.gov, OpenGrey, ISRCTN. Reference lists manually screened. Final search date: 18 March 2025.
	**7**	Present the full search strategies for all databases, registers and websites, including any filters and limits used.	Section 2.3. Information Sources and Search Strategy	Full Boolean search equation for PubMed/MEDLINE provided in-text. Equivalent strings for all other databases available in Supplementary Materials (per manuscript text). No date or language filters applied.
	**8**	Specify the methods used to decide whether a study met the inclusion criteria, including how many reviewers screened each record and whether they worked independently.	Section 2.4. Study Selection	Two reviewers independently screened titles/abstracts then full texts. Disagreements resolved by consensus discussion. Duplicate removal via Zotero (automated + manual). PRISMA flow diagram provided (Figure 2).
	**9**	Specify the methods used to collect data from reports, including how many reviewers collected data from each report and whether they worked independently.	Section 2.5. Data Extraction and Management	Standardized data extraction form used. Two reviewers extracted independently. Discrepancies resolved by mutual agreement. No automated tools used.

	**10a**	List and define all outcomes for which data were sought. Specify whether all results compatible with each outcome domain were sought.	Section 2.2. Eligibility Criteria; Section 2.5	Primary outcomes: oxidative stress biomarkers (MDA, GSH, CAT, SOD, TAC and equivalents). All reported OS biomarkers per study were extracted regardless of direction of effect.
	**10b**	List and define all other variables for which data were sought (e.g. participant and intervention characteristics, funding sources).	Section 2.5. Data Extraction and Management; Table 1	Extracted variables: author, year, design, participant characteristics (sex, n, training status), exercise protocol (modality, intensity, duration), supplement type/dose/duration, and principal findings. Funding not systematically extracted (no meta-analysis planned).
	**11**	Specify the methods used to assess risk of bias in the included studies, including details of the tool(s) used, how many reviewers assessed each study and whether they worked independently.	Section 2.6. Risk of Bias and Methodological Quality Assessment	Cochrane RoB 2.0 tool applied to all 46 included studies across 5 domains. Two reviewers assessed independently; discrepancies resolved by consensus. A supplementary three-tier reporting-completeness classification was also applied (specified/partially specified/not specified). Both tools described in Section 2.6.
	**12**	*Specify for each outcome the effect measure(s) (e.g. risk ratio, mean difference) used in the synthesis or presentation of results.*	*N/A — Not applicable*	*No meta-analysis was conducted due to substantial clinical and methodological heterogeneity across included studies (different supplements, doses, exercise modalities, and OS biomarkers). Results are presented as a qualitative narrative synthesis.*
	**13a**	Describe the processes used to decide which studies were eligible for each synthesis.	Section 2.2; Section 3.1; Table 2	All 46 included studies contributed to the qualitative synthesis. Studies were grouped by supplement category (8 categories; Table 2) based on primary active

				ingredient(s) examined.
	**13b**	Describe any methods required to prepare the data for presentation or synthesis, such as handling of missing summary statistics, or data conversions.	Section 2.5. Data Extraction and Management	No data conversions or imputation were required. All data were extracted directly from published reports. Where biomarker direction was ambiguous, original authors' conclusions were used to classify the study outcome.
	**13c**	Describe any methods used to tabulate or visually display results of individual studies and syntheses.	Table 1; Table 2; Sections 3.2–3.6	Individual study characteristics and outcomes tabulated in Table 1 (46 rows). Supplement category synthesis presented in Table 2. RoB domain results shown in Figures 3A–3D (RoB 2.0 domain charts), Figure 4 (reporting completeness), and Figure 5 (cumulative classification).
	**13d**	*Describe any methods used to synthesise results and provide a rationale for the choice(s). If meta-analysis was performed, describe the model(s) and methods.*	*N/A — Not applicable*	*Meta-analysis was not performed. Narrative synthesis was selected due to: (i) heterogeneity in supplement types (8 categories, 46 studies), exercise modalities (aerobic, resistance, mixed), intervention durations, and OS biomarkers; (ii) absence of a common quantitative outcome measure across studies. This decision is noted in the Strengths and Limitations section.*
	**13e**	*Describe any methods used to explore possible causes of heterogeneity among study results (e.g. subgroup analysis, meta-regression).*	*N/A — Not applicable*	*No formal statistical exploration of heterogeneity (e.g. meta-regression) was conducted as no meta-analysis was performed. Possible moderators (training status, dose, timing, biomarker selection) are discussed narratively in Section 4.1.*
	**13f**	*Describe any sensitivity analyses conducted to assess robustness of the synthesised results.*	*N/A — Not applicable*	*Sensitivity analyses were not applicable given the qualitative synthesis approach. The robustness of conclusions is instead addressed through the RoB 2.0 assessment and*

				GRADE certainty ratings (Sections 2.6–2.7).
	**14**	Describe any methods used to assess risk of bias due to missing results in a synthesis (arising from reporting biases).	Section 2.6; Section 3.4	Reporting completeness was assessed through the supplementary three-tier classification applied in Section 3.4. Funnel plot assessment was not feasible without meta-analysis. Publication bias is acknowledged as a limitation in the Strengths and Limitations section.
	**15**	Describe any methods used to assess certainty (or confidence) in the body of evidence for an outcome.	Section 2.7. Certainty of Evidence	GRADE approach applied. Due to heterogeneity of OS biomarkers across studies, GRADE was applied at supplement-category level (Table 2) rather than for individual outcomes. This limitation is stated in Section 2.1 (PRISMA transparency note).
**RESULTS**	**16a**	Describe the results of the search and selection process, including reasons for exclusions at each stage of the process, and preferably using a flow diagram.	Section 3.1.1; Figure 2	1,444 records identified; 67 duplicates removed; 1,250 records excluded (not investigating the combination); 81 screened by title; 17 manually excluded (reviews n=3; wrong population n=14); 64 screened by abstract; 9 removed; 55 assessed for eligibility; 9 dismissed; 46 included. PRISMA 2021 flow diagram provided as Figure 2.
	**16b**	Cite studies that might appear to meet the inclusion criteria but which were excluded, and explain why they were excluded.	Section 3.1.1; Supplementary Materials	Studies excluded at full-text screening were categorized by reason (reviews, wrong population, wrong outcome, no full text available). Full list of excluded studies with reasons available from the corresponding author on request.
	**17**	Cite each included study and present its characteristics.	Table 1; References [30]–[75]	All 46 included studies are cited (refs [30]–[75]) and their characteristics presented in Table 1:

				study, supplement, design, population size, and overall outcome.
	**18**	Present assessments of risk of bias for each included study.	Sections 3.3–3.5; Figures 3A–3D; Figure 4; Figure 5	Domain-level RoB 2.0 results presented in Figures 3A (randomization), 3B (deviations), 3C (outcome measurement), 3D (selection of results). Reporting-completeness results in Figure 4. Cumulative transparency classification in Figure 5.
	**19**	For all outcomes, present for each study: summary statistics for each group (where appropriate) and an effect estimate and its precision.	Table 1; Sections 3.2.1–3.2.2	Individual study results are presented qualitatively in Table 1 (overall direction of effect per study) and narratively in Sections 3.2.1 and 3.2.2. Quantitative effect estimates and confidence intervals were not extracted as meta-analysis was not performed; this limitation is acknowledged.
	**20a**	For each synthesis, briefly summarise the characteristics and risk of bias among contributing studies.	Sections 3.2; 3.3–3.5; Table 2	Table 2 summarises supplement categories, number of contributing studies, overall evidence trend, and key observations. RoB profile of contributing studies addressed in Section 4.2 (Discussion).
	**20b**	*Present results of all statistical syntheses conducted. If meta-analysis was done, present for each the summary estimate and its precision and measures of statistical heterogeneity.*	*N/A — Not applicable*	*No meta-analysis was performed; no pooled effect estimates or heterogeneity statistics (I^2) are reported. Qualitative synthesis results are presented in Sections 3.2 and Table 2.*
	**20c**	*Present results of any investigations of possible causes of heterogeneity among study results.*	*N/A — Not applicable*	*No formal subgroup analysis or meta-regression was conducted. Possible sources of heterogeneity (training status, dose, timing, biomarker type) are discussed narratively in Section 4.1.*

	**20d**	*Present results of any sensitivity analyses conducted to assess the robustness of the synthesised results.*	*N/A — Not applicable*	*No sensitivity analyses were conducted. Not applicable to qualitative synthesis.*
	**21**	Present assessments of risk of bias due to missing results (arising from reporting biases) for each synthesis assessed.	Section 3.4; Strengths and Limitations	Reporting-completeness assessment is presented in Section 3.4 and Figure 4. Publication bias is acknowledged as a limitation (restriction to English-language publications noted). Formal funnel plot assessment was not feasible without meta-analysis.
	**22**	Present assessments of the certainty (or confidence) in the body of evidence for each outcome assessed.	Section 2.7; Table 2	GRADE certainty assessments are reflected in the "Overall Evidence Trend" column of Table 2 at supplement-category level. Individual outcome-level GRADE ratings were not feasible due to heterogeneity of biomarkers across studies (noted in Section 2.1).
**DISCUSSION**	**23a**	Provide a general interpretation of the results in the context of other evidence.	Section 4.1. Interpretation of Findings	Section 4.1 interprets findings supplement-category by category, discusses moderators (training status, dose, timing, biomarker selection), and contextualises results within the existing polyphenol, omega-3, vitamin, and ergogenic supplement literatures.
	**23b**	Discuss any limitations of the evidence included in the review.	Sections 4.2; Strengths and Limitations	Limitations of the included evidence: small sample sizes, short intervention periods, heterogeneous biomarkers, mixed study designs (RCT and observational), inconsistent reporting, and restriction to English-language publications. All discussed explicitly.
	**23c**	Discuss any limitations of the review processes used.	Strengths and Limitations	Review-process limitations: inability to perform meta-analysis, no sensitivity analysis, qualitative-only

				synthesis, language bias (English only), and the use of a transparency-based rather than purely design-based RoB classification. All discussed.
	24	Provide a general interpretation of the results in the context of other evidence and implications for future research.	Sections 4.3–4.5; Section 5 (Conclusions)	Broader implications for clinical, sports, and rehabilitation practice discussed in Section 4.3. Future research directions (standardized protocols, dose–response, long-term RCTs) outlined in Section 4.4. Conclusions in Section 5.
	25	Provide a general interpretation of the results with implications for future research.	Section 5. Conclusions	Three-paragraph conclusion: (1) summary of supplement-category findings; (2) methodological weaknesses and their implications for confidence; (3) call for standardized, long-term RCTs specifying exercise modality and intensity with explicit dose–response characterization.
OTHER INFORMATION	26a	Provide registration information for the review, including register name and registration number, or state that the review was not registered.	Section 2.1. Study Design and Registration	Prospectively registered in PROSPERO. Registration ID: CRD42024014399. Stated in Section 2.1.
	26b	Indicate where the review protocol can be accessed, or state that a protocol was not prepared.	Section 2.1	Protocol available via PROSPERO (CRD42024014399). The PROSPERO registration record constitutes the prospectively stated protocol for this review.
	26c	Describe and explain any amendments to information provided at registration or in the protocol.	Section 2.1	No amendments were made to the registered protocol during the conduct of this review. The review was completed as prespecified.
	27	Describe sources of financial or other support for the review, and the role of the funders or sponsors in the review.	Funding statement (backmatter)	Stated in the Funding section: "This research received no external funding."

	**28**	Declare any competing interests of review authors.	Conflicts of Interest statement (backmatter)	Stated in the Conflicts of Interest section: "The authors declare no conflicts of interest."
	**29**	Report which data, analytic methods, and/or other materials used in the review are publicly available and where they can be found.	Data Availability Statement (backmatter)	Stated in the Data Availability Statement: data derived from published studies; no new datasets generated; extracted data available from corresponding author on request.

Legend: Items highlighted in yellow are Not Applicable (N/A) to this review because no meta-analysis was conducted. All other PRISMA 2021 items are addressed in the manuscript. Items 12, 13d–f, 20b–d: require quantitative synthesis; N/A. Item 15: applied at category level (see Section 2.7 and note in Section 2.1).
